# Findings from a cluster randomised trial of unconditional cash transfers in Niger

**DOI:** 10.1111/mcn.12615

**Published:** 2018-05-08

**Authors:** Victoria L. Sibson, Carlos S. Grijalva‐Eternod, Garba Noura, Julia Lewis, Kwanli Kladstrup, Hassan Haghparast‐Bidgoli, Jolene Skordis‐Worrall, Tim Colbourn, Joanna Morrison, Andrew J. Seal

**Affiliations:** ^1^ Institute for Global Health University College London London UK; ^2^ Concern Worldwide Niamey Niger

**Keywords:** child health, child nutritional status, emergencies, household food security, malnutrition, unconditional cash transfers

## Abstract

Unconditional cash transfers (UCTs) are used as a humanitarian intervention to prevent acute malnutrition, despite a lack of evidence about their effectiveness. In Niger, UCT and supplementary feeding are given during the June–September “lean season,” although admissions of malnourished children to feeding programmes may rise from March/April. We hypothesised that earlier initiation of the UCT would reduce the prevalence of global acute malnutrition (GAM) in children 6–59 months old in beneficiary households and at population level. We conducted a 2‐armed cluster‐randomised controlled trial in which the poorest households received either the standard UCT (4 transfers between June and September) or a modified UCT (6 transfers from April); both providing 130,000 FCFA/£144 in total. Eligible individuals (pregnant and lactating women and children 6–<24 months old) in beneficiary households in both arms also received supplementary food between June and September. We collected data in March/April and October/November 2015. The modified UCT plus 4 months supplementary feeding did not reduce the prevalence of GAM compared with the standard UCT plus 4 months supplementary feeding (adjusted odds ratios 1.09 (95% CI [0.77, 1.55], *p* = 0.630) and 0.93 (95% CI [0.58, 1.49], *p* = 0.759) among beneficiaries and the population, respectively). More beneficiaries receiving the modified UCT plus supplementary feeding reported adequate food access in April and May (*p* < 0.001) but there was no difference in endline food security between arms. In both arms and samples, the baseline prevalence of GAM remained elevated at endline (*p* > 0.05), despite improved food security (*p* < 0.05), possibly driven by increased fever/malaria in children (*p* < 0.001). Nonfood related drivers of malnutrition, such as disease, may limit the effectiveness of UCTs plus supplementary feeding to prevent malnutrition in this context. Caution is required in applying the findings of this study to periods of severe food insecurity.

**Key messages**

- Starting the UCT earlier and providing the same amount of cash over 6 months instead of 4, alongside 4 months supplementary feeding, temporarily increased beneficiary food security, but did not impact on children's nutritional status at end line.
- GAM prevalence remained static and elevated, despite improved food security; probably due to a deteriorating health situation.
- Strengthening interventions to tackle malaria, as well as providing seasonal cash and food, may better protect children from acute malnutrition.
- Future studies should test combined health and food security interventions and explore the assumption that the targeting of low income households leads to population level impact.

## 1 INTRODUCTION

Wasting (defined as weight for height < −2 Z scores) accounts for 14.6% of mortality among children under 5 years old (McDonald et al., 2013). Nearly a quarter of the world's nations have a prevalence of acute malnutrition (defined as weight for height < −2 Z scores or nutritional oedema) of at least 10% (United Nations Childrens' Fund et al., 2012), which is an “emergency requiring immediate intervention” (World Health Organization, 2000). The highest prevalence of acute malnutrition is in south Asia and sub‐Saharan Africa (United Nations Childrens' Fund, World Health Organization, & The World Bank, 2012), regions with frequent shocks such as natural disasters (Centre for Research on the Epidemiolgy of Disasters, 2015) and complex emergencies (Spiegel, Le, Ververs, & Salama, 2007), which often require humanitarian assistance.

Effective prevention of acute malnutrition requires addressing its causes, including food insecurity, deficiencies in the social/care environment, disease, and inadequacies in environmental health. However, in many humanitarian settings, food‐based approaches such as general food distributions and supplementary feeding, predominate (Bailey & Hedlund, 2012) despite limited evidence of effectiveness (Sguassero, Onis, Bonotti, & Carroli, 2012), implementation challenges (Hall, Oirere, Thurstans, Ndumi, & Sibson, 2011; US Centers for Disease Control and Prevention, 2013) and concerns about cost‐effectiveness (Puett et al., 2013). These limitations have contributed to an increase in short‐term Unconditional Cash Transfer (UCT) and voucher interventions (Harvey, Proudlock, Clay, Riley, & Jaspars, 2010; Overseas Development Institute, 2015; World Food Programme, 2010). However, although cash/vouchers have the potential to address multiple causes of malnutrition in contexts with functional markets and adequate supply (Leroy, Ruel, & Verhofstadt, 2009), evidence for nutritional impact in humanitarian contexts is inconclusive. Most studies have focused on linear growth among beneficiaries of longer‐term Conditional Cash Transfers (CCTs) in development contexts (Manley, Gitter, & Slavchevska, 2013; Bastagli et al., 2016). A recent review of 14 studies of CCTs and UCTs in low and middle income countries that assessed anthropometry found that 9 had no impact on anthropometric outcomes. Furthermore, only five measured wasting and of these, only one (a CCT in a mixed rural/urban setting in Bangladesh) documented significant improvement (Bastagli et al., 2016).

The strongest evidence of the nutrition‐related effect of cash transfers is on food security (Arnold, Conway, & Greenslade, 2011; Bastagli et al., 2016; de Groot et al., 2015; Fiszbien & Schady, 2009; Manley et al., 2013), but there remains a lack of robust studies of impact on health (Bailey & Hedlund, 2012; Pega, Liu, Walter, & Lhachimi, 2015) and the care determinants of child nutrition (Bailey & Hedlund, 2012; de Groot et al., 2015; Quisumbing & McClafferty, 2006; Schady & Rosero, 2008). Evidence from multiyear CCTs has shown they can improve uptake of health services where these are available (Bastagli et al., 2016; de Groot et al., 2015; Fiszbien & Schady, 2009) and improve hygiene practices (de Groot et al., 2015). However, these do not often translate into improved health or nutrition outcomes (Fiszbien & Schady, 2009). In humanitarian settings in particular, cash is unlikely to impact on health unless access to quality services is only limited by poverty (Bailey & Hedlund, 2012).

Given the consensus that cash transfers are unlikely to affect nutritional status when implemented in isolation (Bailey & Hedlund, 2012; Bhutta et al., 2013; de Groot et al., 2015), studies in contexts prone to shocks have mostly tested the effect of cash combined with supplementary feeding or nutrition‐related education, finding combinations more effective than cash or food alone (Ahmed, Quisumbing, Nasreen, Hoddinott, & Bryan, 2013; Langendorf et al., 2014). The lack of compelling evidence regarding the impact of cash transfers on nutrition‐related indicators is likely due to differences in programmatic context, implementation, and design (Bastagli et al., 2016), including targeting, amount, duration, and timing (Arnold et al., 2011; Manley et al., 2013). More studies are therefore required to build the evidence base on where, when, and how cash‐based interventions are effective against acute malnutrition.

Seasonal UCTs have been implemented by international non‐governmental organisations in Niger since 2008 with humanitarian funding to coincide with the preharvest lean season. However, there is inconclusive evidence of their nutritional impact and questions about how to optimise their design remain (Aker, Boumnijel, McClelland, & Tierney, 2011; Aker & Nene, 2012; Bliss et al., 2016; Bliss & Golden, 2013; Fenn, Noura, Sibson, Dolan, & Shoham, 2014; Langendorf et al., 2014; Poulsen & Fabre, 2011; Save the Children, 2009). Furthermore, monthly trends in the admission of acutely malnourished children to feeding programmes indicate that acute malnutrition incidence may not coincide with the lean season, as admissions may rise before and/or after (Figure S1.1). There was also an absence of studies on the nutritional impact of this targeted intervention at the population level, despite the assumption of operational agencies that socio‐economic targeting will prevent rising acute malnutrition prevalence.

We tested whether starting the UCT 2 months earlier, but providing the same total amount of cash over 6 months instead of 4, alongside supplementary food for pregnant and lactating women and children 6–<24 months between June and September, would reduce the prevalence of acute malnutrition in children in targeted households and in the general population. The study protocol was published in 2015 (Sibson et al., 2015).

## 2 PARTICIPANTS AND METHODS

### 2.1 Ethics

The trial (ISRCTN 25360839) was approved by the Comité Consultatif National d'Ethique in Niger (ID number 021/2014/CCNE) and University College London (project ID 6543/001). Informed written consent was obtained from all participants.

### 2.2 Setting

The study setting was the rural communes of Affala and Takanamatt in the department of Tahoua, southwest Niger. Hausa is the largest ethnic group, followed by Tuareg and Fulani. The sedentary, agro‐pastoral communities rely on the single, unpredictable rainy season between June and September. This is also the “lean” season, when stores of crops harvested the previous year begin to run out and the prices of goods with lower availability increase in the market (Figure S1.2). Rain‐fed millet, sorghum, and cow peas are harvested September–November. However, most households produce insufficient cereals for subsistence, and also undertake daily labour, labour migration, petty trade, and borrow and sell assets to maintain food access (Anonymous, 2012). Livestock holdings are small, typically a few sheep/goats, fowl, and a donkey for the poorest, whereas the better‐off may own cattle and/or camels (Anonymous, 2012). Besides food insecurity, challenges in the public health, social, and caring environments also exist. There is a chronic shortage of water in most areas, poor hygiene practices, and few latrines (Hampshire, Casiday, Kilpatrick, & Panter‐Brick, 2009). Malaria is endemic during the rains (Blanford, Kumar, Luo, & MacEachren, 2012) and diarrhoea and acute respiratory infection (ARI) rates are also high (Hampshire, Casiday, et al., 2009). Despite a policy of free health care for children under 5 and pregnant women, there is low utilisation due to facility inaccessibility (Blanford et al., 2012) and household prioritisation of livelihood preserving activities over health‐seeking (Hampshire, Panter‐Brick, Kilpatrick, & Casiday, 2009). Nutrition surveys of Tahoua indicate that the Global Acute Malnutrition (GAM) prevalence (weight for height Z‐score (WHZ) <−2 (WHO 2006 growth standards) and/or oedema) consistently remains concerning (>10%; World Health Organization, 2000) during the lean season and/or post‐rains/harvest.

Since 2006, the international non‐governmental organisations Concern Worldwide (hereafter Concern) has been working with the government in the communes of Affala and Takanamatt to prevent and treat acute malnutrition. Concern started implementing livelihoods and water, sanitation and hygiene programmes in 2009 and began annual, seasonal UCTs in 2010.

### 2.3 Outcomes

Using a cluster‐randomised controlled trial study design, we assessed impact both among children in beneficiary households and in the general population (i.e., a representative sample of beneficiaries and nonbeneficiaries), hypothesising that the earlier UCT would be more effective at preventing acute malnutrition in both populations. The primary outcome was the prevalence of GAM in children aged 6–59 months. Secondary outcomes reported here include prevalence of mid‐upper arm circumference (MUAC) <12.5 cm and/or oedema in children 6–59 months (which we report as prevalence of low MUAC only because of the small number of oedema cases); mean WHZ (WHO 2006 growth standards) in children 6–59 months; mean MUAC in children 6–59 months; and mean household expenditure, household dietary diversity score, and individual dietary diversity score in children 6–59 months (which we report for children 6–23 and 24–59 months separately, because the former age group is that used for internationally recommended IYCF indicators and there is no internationally agreed indicator for the latter). Testing of hypotheses with anthropometric outcomes was conducted using adjusted regressions models, whereas a difference in difference approach was used to describe changes in potential mediating factors and nonanthropometric secondary outcomes. We also conducted a process evaluation to describe the context and fidelity of intervention implementation, to aid interpretation of the results and assess generalisability. This involved the collation of routinely collected programme and health facility M&E data, interviewing key programme staff and conducting field visits.

### 2.4 Population

Our main study population of interest was children, aged 6–59 months, living in villages that had been selected to receive the UCT by humanitarian targeting criteria. All children in this age range and living in these villages were eligible for inclusion. We exhaustively sampled all beneficiary households in the targeted villages (*n* = 2,073), and included all children 6–59 months within. We used simple random sampling (probability proportional to cluster size) to sample 500 nonbeneficiary households in these same villages in which we also exhaustively sampled children 6–59 months old. We used Concern's village‐level household census and calculated an average of 1.79 eligible children per household. We collected household level data whether or not the household had any eligible children.

Eligibility for the UCT was determined by Concern according to their usual three‐step procedure: (a) selection of villages that had received cash in 2014 and/or 2013 and had a forecasted production deficit in 2015; (b) wealth ranking using household survey data (drawing on the principles of the Household Economy Approach that categorises households as “very poor,” “poor,” “middle,” and “better off”; Boudreau et al., 2008); and (c) selection of all the very poor and as many of the poor as possible, until funds were fully allocated. The poorest households typically had less than 10 members, possessed nine chickens, five sheep/goats, no large ruminants, cultivated fewer than 4 ha, forecast fewer than 5 months yield from their last harvest and earned less than 35,000FCFA/month. This led to the UCT being targeted to 42% of households in the study villages. Concern's household definition was a group of people preparing and eating food together. There were no exclusion criteria for households but we excluded disabled children.

### 2.5 Randomisation

The unit of randomisation was a cluster of villages assigned to receive UCT at the same cash distribution point (CDP), which was within 5 km of the village. We used a clustered design because the intervention was delivered at the village level. There were 25 CDPs in total and to ensure reasonable balance in sample size between arms, we grouped CDP serving smaller populations into a single cluster prior to randomisation. We also limited cluster size by excluding the four largest villages prior to randomisation. This resulted in 20 clusters containing 39 villages. Clusters were randomised in a 1:1 ratio to either the “standard” (June UCT initiation) or the “modified” (April UCT initiation) intervention. The randomisation was undertaken in a public meeting of village leaders managed by the study coordinator. The names of the villages in each cluster were written on papers, one paper per cluster, and placed into identical envelopes that participants blindly selected one‐by‐one for sequential allocation of clusters to the two arms.

### 2.6 Interventions

The standard UCT intervention consisted of a monthly transfer of 32,500FCFA (equivalent to £36), for 4 months between June and September 2015; total 130,000FCFA (equivalent to £144). The transfer was designed to allow purchase of a food basket similar to the World Food Programme household ration (cereal, pulse, and vegetable oil) that would meet 75% of the daily energy needs of a seven‐person household. The modified UCT intervention consisted of 21,500FCFA/month (£24) in April, May, July, August, and September and 22,500FCFA in June; total also 130,000CFA. In both arms, cash‐in‐hand was given to female household representatives. At each distribution beneficiaries first had to attend an education session, which included suggestions on buying food for children, following which women and children were screened for acute malnutrition and provided referral slips as necessary. After this, beneficiaries received their cash. In addition, between June and September, beneficiary households in both study arms were also given 200 g/day Super Cereal Plus for each child 6–<24 months (providing 820 kcal/day) and 250 g/day Super Cereal and 75 g/day vegetable oil for each pregnant/lactating woman (providing 1,613 kcal/day).

### 2.7 Sample size

The required sample of beneficiary children, aged 6–59 months, was calculated using the *clustersampsi* command in Stata (Hemming & Marsh, 2013), to allow detection of a difference in endline GAM prevalence between the arms of 7% points. Based on previous data from the area, we assumed an average cluster size of 176 children with 1.8 children per household, a baseline GAM prevalence of 21% (Bliss & Golden, 2013), an ICC = 0.0138, and allowed for a 5% Type 1 error risk and 80% power. The total calculated sample size required was 3,520 children in 1,956 households, in 20 clusters. A sample of 500 nonbeneficiary households was determined pragmatically, being the maximum additional number of households we could enumerate with our available resources. This sample was allocated between clusters according to the number of households in each cluster. During data collection, 100% of beneficiary households and 17.6% of nonbeneficiary households were sampled from each cluster.

### 2.8 Data collection

We collected quantitative data in March–April 2015, before the intervention (baseline), and in October–November 2015, after the intervention (endline), using structured questionnaires in Samsung Galaxy G2 7.0‐inch tablets running PSI Fusion software. We trained study staff over 2 weeks before both rounds, including anthropometry standardisation tests. Data were collected at the respondents' homes. Children's anthropometric measurements (weight, height, MUAC) were taken and recorded twice and presence/absence of oedema checked at each time point. Weight was measured to 100 g using an electronic scale (SECA model 870). Length in children <24 months and height in children ≥24 months were measured to 1 mm using a stadiometer (Infant/child/adult ShorrBoard). MUAC was measured on the left arm using a TALC‐UK insertion tape to 1 mm. Peripheral blood was collected from a finger prick using a safety lancet and haemoglobin concentration was assessed to 0.1 g/dl precision, using a portable photometer HemoCue® 301 analyser. Acutely malnourished and/or anaemic children were referred for treatment. Unfortunately, we are not able to report the results of the haemoglobin analysis in this paper due to concerns about the reliability of the data. Four‐week retrospective morbidity among children was collected by caregiver recall, for a range of common symptoms and/or diseases.

In addition to primary data collection, we compiled Concern's routine quantitative monitoring data and undertook interviews with programme staff for the process evaluation. More specifically on the evaluation of intervention implementation, we used monitoring data made available by Concern that described accessibility, acceptability, timeliness, and coverage. Data on accessibility and acceptability came from distribution monitoring undertaken by Concern staff on the day of distribution (between June and September only), in which a small randomly selected sample of beneficiary women were administered a short questionnaire. Data on timeliness came from interviewing the deputy programme manager. Data on coverage came from monthly programme implementation reports.

### 2.9 Data processing and analysis

We used Stata version 14 (StataCorp, 2015) for data management and analysis. Thirty‐day expenditure was estimated by adding all recalled spends for the last month (mostly food stuffs) and the average spend over 30 days on additional items such as rent and health care, calculated from recalled spends made during the previous 6 months. An asset index was created using principal components analysis (Vyas & Kumaranayake, 2006) using a list of 33 assets considered neither too rare nor too common; for this we used a 5% cut‐off value; for example, >95% of households had access to land so this was too common a commodity to be included in the index. The household food insecurity access scores, reduced coping strategies index scores, food consumption scores, household dietary diversity score, and months of adequate household food provisioning were calculated using standard procedures (Bilinsky & Swindale, 2007; Coates, Swindale, & Bilinsky, 2007; Maxwell & Caldwell, 2008; Swindale & Bilinsky, 2006; World Food Programme, 2008). Improved water sources included piped water, taps, boreholes, protected wells, and rain water. Improved latrines included flush to septic system or pit, pit, and composting latrines. Infant and young child feeding practice indicators were calculated using standard definitions (World Health Organization, 2008), with the exception of the dietary diversity score for children 24–59 months, for which we used seven food groups: grains; roots and tubers; legumes and nuts; dairy products; flesh foods; eggs; vitamin A‐rich fruits and vegetables; other fruits and vegetables. Child's age at endline was derived from birth date or estimated using a local calendar of events. Age at baseline was estimated as endline age minus the follow‐up period because of better endline data reliability. The anthropometric indices, WHZ and height for age Z‐score (HAZ), were calculated using the *zanthro* command (Vidmar, 2013) and flagged values were excluded from analysis according to the cut‐offs: WHZ <−5 and >5 (*n* = 5) and HAZ <−6 and >6 (*n* = 25; Crowe, Seal, Grijalva Eternod, & Kerac, 2014). Further exclusions of height, WHZ, and HAZ data were made on the basis of implausible longitudinal gains in height of <0.00 (*n* = 50) or ≥15.00 cm (*n* = 9). The latter value was used as the +3 Z‐score length increase in children aged 6–18 months over 6 months in the reference population is 11.9 cm (World Health Organization, 2016). For MUAC, the following baseline measurements were excluded from analysis for being biologically implausible: 33 mm (*n* = 1); ≥125 mm from participants with WHZ <−4 WHZ (*n* = 3); <125 mm from participants with WHZ ≥1 (*n* = 2); and at endline measurements ≥125 mm from participants with WHZ <−4 (*n* = 2). Examination of longitudinal MUAC change indicated one additional value (−96 mm) to exclude.

The primary outcome, endline GAM prevalence in children 6–59 months old at baseline, was analysed at individual level using mixed effects multilevel logistic regression, accounting for variation at the cluster level using random effects. Covariates included baseline WHZ, sex, and age and those found to differ between arms at baseline (tested using chi square/*t* tests on transformed data as necessary). We tested for baseline differences between arms because we anticipated that the small number of clusters would produce some significant variation by chance (Hayes & Moulton, 2009). Secondary nutrition outcomes at the individual child level were also analysed using adjusted mixed effects multilevel regressions. We did investigate whether commune had any predictive value for our outcomes and it did not. We did not collect village level data because of resource constraints but we do not think that this would have impacted on the results. Endline differences between arms in possible determinants of undernutrition were analysed using a difference in differences approach, with the exception of infant and young child feeding practices for which the age‐limited groups for each indicator rendered difference in difference analysis impossible. Instead, to test for any differences in these variables at endline, we performed comparative cross‐sectional analyses. It should be noted that predictors and outcome measures were analysed using different approaches because the odds ratio allowed for an adjusted model to be constructed to test the impact of the intervention on the outcome, whereas the difference in differences provided a more descriptive and understandable way to look at potential changes in mediating variables.

For population level analyses, the samples of beneficiaries and nonbeneficiaries were combined. To account for the relative undersampling of nonbeneficiaries and ensure that the results were representative of the general population, a population weight of 5.68 was calculated and applied to nonbeneficiaries.

## 3 RESULTS

### 3.1 Intervention implementation and uptake

Typically, the targeted female recipient attended the cash distribution, travelling under 1 hr and rarely incurring expense. Distributions started on April 14 and ended on September 16, 2015, and although most were timely, some in July and August were 3 to 5 days late. The uptake of cash and supplementary food by beneficiary households, that is, the collection of the item from the distribution point, was close to 100%.

More specifically, there were 1,130 beneficiary households targeted with the standard intervention. Four households were dropped by the August distribution because they could not be traced. Absence or other reasons for nonreceipt affected between 1 and 12 intended beneficiaries each month; that is, coverage was between 98% and 99% over the 4 months of distribution.

There were 963 beneficiary households targeted with the modified intervention. Three households were dropped for the May distribution, including one double registered. Absence or other reasons for nonreceipt affected only between 0 and 6 intended beneficiaries each month; that is, coverage was between 99% and 100% over the 6 months of distribution. The small difference in the number of programme beneficiaries and our samples was likely due to the double registration of a small number of additional households.

Reasons for nonreceipt included the beneficiary attending a funeral; travelling to work on the land far from the CDP; losing their card; delivering a baby or being hospitalised; or caring for someone else sick. Beneficiaries missing a distribution were able to collect double cash in the next month's distribution.

There were no discernible differences in implementation or uptake between arms.

### 3.2 Participant flow

**Figure 1:**
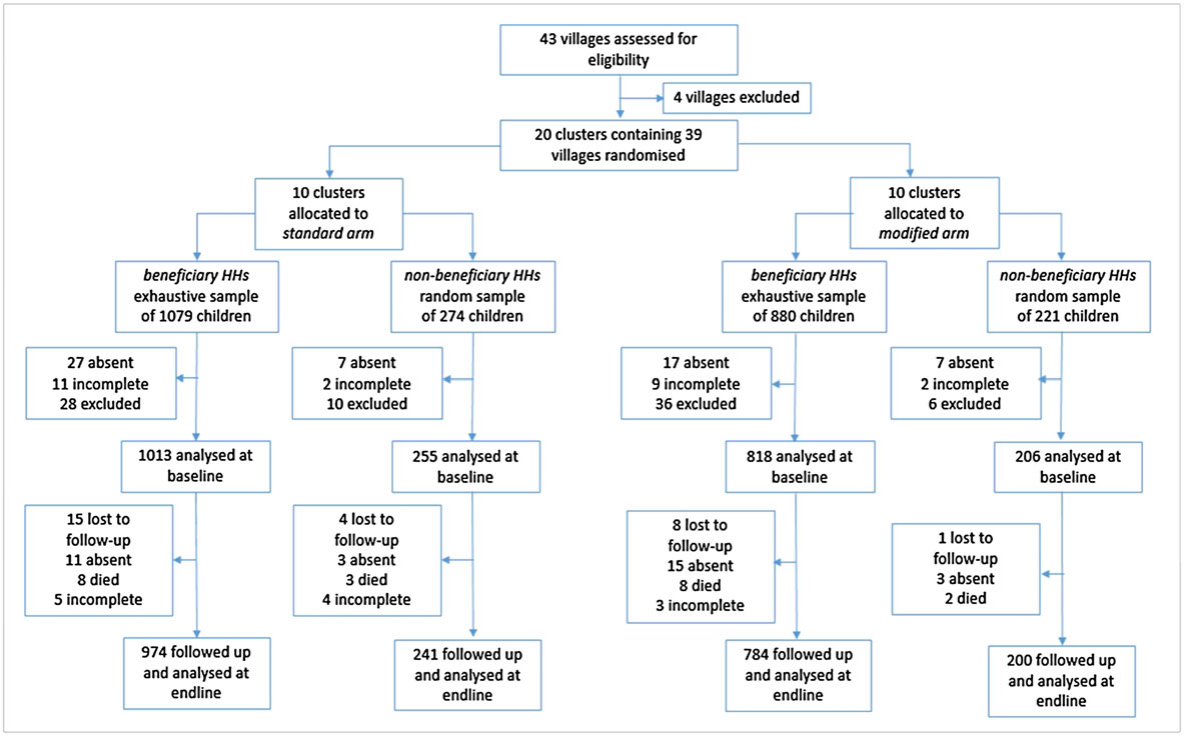
Flow diagram of child trial participants

We undertook baseline data collection in March–April 2015. All beneficiary households living within the standard and modified study arms were recruited (1,124 and 949, respectively). From these households, we sampled all 1,959 eligible children and obtained baseline measures from 1,831 (Figure 1). In addition, we sampled 495 children from nonbeneficiary households within both study arms and successfully obtained baseline measurements from 461. The overall baseline participant response rate was 93%.

We undertook endline data collection in October–November 2015. Of those children with baseline data, 28 were lost to follow‐up, 32 were absent, 21 died, and 12 had incomplete data. The overall follow‐up proportion in both arms was 96.0%. Mean follow‐up for beneficiary children was 213 days (*SD* = 4.5, 95% CI [212, 214], range 202–254), with no difference between arms (*p* = 0.280) and the same for the population sample (213 days (*SD* = 4.5, 95% CI [212, 215], range 202–254), (*p* = 0.357)). See Figure S2.1 for a flow diagram of households.

### 3.3 Baseline characteristics

#### 3.3.1 Beneficiaries

The average household comprised five people, including 1.5 children 6–59 months old (1.50 children in the standard arm and 1.42 in the modified arm; 1,831 children in 1,254 households; Table 1). Two thirds of households had at least one child 6–59 months old. The majority ethnicity was Hausa with a third Tuareg. Three quarters of households classified themselves as male‐headed, whether or not the head was currently living at home. Most were sedentary, with a minor but significantly greater proportion of nomads and transhumant lifestyles (seasonal migration with animals for pasture) in the standard arm. Most households had land access; significantly more in the standard arm. The reported mean number of livestock owned was low in both arms, but with wide ranges. Average 30‐day household expenditure was equivalent to £29, with no difference between arms; less than the standard UCT (32,500FCFA or £36/month). However, the wide range (£0–142/month) indicates some inequality and possible targeting errors that are consistent with the wide range in livestock holdings. A range of indicators suggest relative food security in both arms at baseline, but low average dietary diversity. In both arms, very few households had access to improved latrines, but two thirds had access to an improved water source. A comparison of beneficiaries and nonbeneficiaries will be reported elsewhere.

**Table 1:** Baseline characteristics of beneficiary households

Characteristic		Standard arm (June initiation)	Modified arm (April initiation)	Combined arms	*P* value
Households (*n*)		1,040	895	1,935	
*Sociodemographic characteristics*					
Number in HH (mean ± *SD*)		5.4 ± 2.6	5.3 ± 2.4	5.4 ± 2.5	0.314
Ethnicity of HH head (*n* [%] 95% CI)	Hausa	772 (74.2) (58.9, 85.3)	470 (52.5) (34.4, 70.0)	1,242 (64.2) (50.4, 76.0)	0.065
	Tuareg	249 (23.9) (13.4, 39.2)	394 (44.0) (27.3, 62.3)	643 (33.2) (22.0, 46.7)	
	Fulani/Peulh	16 (1.5) (0.8, 3.0)	30 (3.4) (2.1, 5.3)	46 (2.4) (1.5, 3.7)	
	Other	3 (0.3) (0.1, 0.8)	1 (0.1) (0.0, 0.7)	4 (0.2) (0.1, 0.5)	
Sex of HH head (*n* [%] 95% CI)	Male	801 (77.0) (69.3, 83.3)	653 (73.0) (64.8, 79.8)	1454 (75.1) (69.4, 80.1)	0.420
Lifestyle of HH (*n* [%] 95% CI)	Sedentary	922 (88.7) (80.4, 93.7)	867 (96.9) (94.5, 98.2)	1789 (92.5) (87.0, 95.7)	0.002
	Nomad	57 (5.5) (2.3, 12.3)	24 (2.7) (1.4, 5.2)	81 (4.2) (2.2, 7.9)	
	Transhumant/other	61 (5.9) (3.7, 9.2)	4 (0.5) (0.2, 1.2)	65 (3.4) (1.8, 6.2)	
*Wealth*					
30‐day expenditure (GBP equivalent, mean ± *SD*, range)		28.40 ± 17.66 (0–141.87)	29.00 ± 19.17 (0.55–131.87)	28.67 ± 18.38 (0–141.87)	0.679
Access to land (*n* (%) 95% CI)		999 (96.1) (94.8, 97.0)	833 (93.1) (89.7, 95.4)	1832 (94.7) (92.9, 96.0)	0.025
Large ruminants owned (mean ± *SD*, range)		0.5 ± 0.8 (0–8)	0.7 ± 1.0 (0–13)	0.6 ± 0.9 (0–13)	0.141
Small ruminants owned (mean ± *SD*, range)		1.7 ± 2.4 (0–30)	2.1 ± 2.5 (0–18)	1.9 ± 2.5 (0–30)	0.087
*Food security*					
Household food insecurity access score (mean ± *SD*) (0 best–27 worst)		7.4 ± 5.5	7.3 ± 5.6	7.3 ± 5.	0.945
Coping strategies index score (mean ± *SD*) (0 best–56 worst)		8.3 ± 9.4	8.6 ± 9.5	8.5 ± 9.4	0.602
7‐day food consumption score (mean ± *SD*) (>35 “acceptable”)		40.9 ± 18.0	43.7 ± 16.9	42.2 ± 17.6	0.329
24‐hr household dietary diversity score^a^ (mean ± *SD*) (min 0–max 12)		4.0 ± 2.0	4.2 ± 1.9	4.1 ± 2.0	0.448
*Sanitation and hygiene*					
Use an improved water source (*n* (%) 95% CI)		765 (73.6) (56.2, 85.8)	519 (58.0) (50.0, 65.5)	1284 (66.4) (55.3, 75.9)	0.094
Use an improved latrine (*n* (%) 95% CI)		18 (1.7) (0.5, 6.0)	24 (2.7) (0.8, 8.2)	42 (2.2) (0.9, 5.1)	0.595

a Denominator for modified arm: 894.

There were no differences between arms in average child age or sex ratio (Table 2). Over 10% of children were wasted at baseline and, when combined with the small number of oedema cases, the GAM prevalence was 13.5% (14.1% standard arm, 12.9% modified arm, *p* = 0.685); that is, at emergency levels (World Health Organization, 2000). The prevalence of low MUAC was small, and significantly greater in the standard arm, whereas the mean WHZ and HAZ scores were below zero and over a third of children were stunted. In addition, nearly 1/3 children were sick in the previous month, of which more than half had fever/malaria and the rest had ARI or diarrhoea. ARI was more common among beneficiary children in the modified arm. Bed net use was very low, as may be expected given the hot/dry conditions with few mosquitoes. Most of the children under 2 years old had been breastfed and a similar proportion of 12–15 month olds were still being breastfed. However, very few children were receiving a minimum adequate diet or achieving minimum dietary diversity or meal frequency. Children 24–59 months of age consumed only 2/7 food groups on average the previous day, which is consistent with the poor household dietary diversity.

**Table 2:** Baseline characteristics of beneficiary children

Characteristic	Standard arm (June initiation)	Modified arm (April initiation)	Combined arms	*P* value
Children (*n*)	1,013	818	1,831	
Male sex (*n* (%) 95% CI)	504 (49.8) (46.8, 52.7)	411 (50.2) (45.1, 55.4)	915 (50.0) (47.2, 52.8)	0.864
Age (months, mean ± *SD*)	29.1 ± 13.7	29.8 ± 14.1	29.4 ± 13.9	0.263
*Nutrition status*				
WHZ (mean ± *SD*)^a^	−0.98 ± 1.00	−0.87 ± 0.99	−0.93 ± 1.00	0.252
Wasted (<−2 WHZ, *n* (%) 95% CI)^a^	134 (13.8) (9.9, 18.8)	99 (12.7) (9.2, 17.2)	233 (13.3) (10.5, 16.7)	0.703
Global acute malnutrition (<−2 WHZ and/or oedema, *n* (%) 95% CI)^b^	137 (14.1) (10.3, 18.8)	101 (12.9) (9.5, 17.4)	236 (13.5) (10.8, 16.8)	0.685
MUAC (mm, mean ± *SD*)^c^	142 ± 13	144 ± 12	143 ± 12	0.045
Low MUAC (<125 mm, *n* (%) 95% CI)^c^	73 (7.3) (5.9, 9.0)	31 (3.8) (2.6, 5.6)	104 (5.7) (4.4, 7.4)	0.005
HAZ (mean ± *SD*)^d^	−1.49 ± 1.44	−1.44 ± 1.26	−1.47 ± 1.37	0.571
Stunted (<−2 HAZ, *n* (%) 95% CI)^d^	355 (36.6) (33.0, 40.3)	271 (34.8) (30.6, 39.3)	626 (35.8) (33.0, 38.7)	0.537
*Infection and health behaviour* e				
Sick in previous 4 weeks (*n* (%) 95% CI)	279 (27.5) (18.0, 39.8)	257 (31.5) (26.8, 36.5)	536 (29.3) (23.1, 36.4)	0.516
Sick with fever/malaria (*n* (%) 95% CI)	161 (57.7) (51.8, 63.4)	137 (53.3) (45.7, 60.7)	298 (55.6) (50.6, 60.5)	0.347
Sick with ARI (*n* (%) 95% CI)	63 (22.6) (16.3, 30.4)	90 (35.0) (28.7, 41.9)	153 (28.5) (23.0, 34.8)	0.017
Sick with diarrhoea (*n* (%) 95% CI)	52 (18.6) (14.6, 23.5)	49 (19.1) (15.3, 23.5)	101 (18.8) (16.0, 22.1)	0.884
Slept under a mosquito net night before (*n* (%) 95% CI)	99 (9.8) (6.8, 13.8)	72 (8.8) (5.8, 13.2)	171 (9.3) (0.7, 12.2)	0.696
*Care/nutrient intake*				
Children 6–<24 months	393	311	704	
Ever breastfed (*n* (%) 95% CI)	341 (86.8) (81.8, 90.5)	261 (83.9) (77.1, 89.0)	602 (85.5) (81.4, 88.8)	0.420
Continued breastfeeding at 1 year (*n* (%) 95% CI)^f^	73 (83.0) (73.0, 90.2)	63 (84.0) (69.4, 92.4)	163 (83.4) (75.1, 89.4)	0.882
Minimum dietary diversity (*n* (%) 95% CI)	63 (16.0) (10.8,23.2)	67 (21.5) (18.0,25.6)	130 (18.5) (14.5, 23.2)	0.152
Minimum meal frequency (*n* (%) 95% CI)	81 (20.6) (15.0, 27.7)	70 (22.5) (17.7, 28.2)	151 (21.5) (17.5, 26.0)	0.637
Minimum adequate diet (*n* (%) 95% CI)	13 (3.3) (1.4, 7.5)	18 (5.8) (3.5, 9.5)	31 (4.4) (2.6, 7.3)	0.238
Children 24–<59 months	620	507	1,127	
24‐hr seven food group diet diversity score (mean ± *SD*)	2.3 ± 1.3	2.5 ± 1.3	2.4 ± 1.3	0.437

*Note*. WHZ = weight for height Z‐score; MUAC = mid‐upper arm circumference; HAZ = height for age Z‐score; ARI = acute respiratory infection.

a Denominator for standard arm: 972, and for modified arm: 780.

b Denominator for standard arm: 975, and for modified arm: 782; there were five oedema cases in total, three in the standard arm and two in the modified arm.

c Denominator for standard arm: 1003, and for modified arm: 809.

d Denominator for standard arm: 971, and for modified arm: 778.

e Denominator for modified arm: 817.

f Estimated for children 12–15 months only; denominator for standard arm: 88, and for modified arm: 75.

#### 3.3.2 Population sample

Baseline characteristics of the population sample were similar to the beneficiaries (Tables S1 and S2), due in part to sample overlap. The differences between arms were proportions of households by lifestyle (fewer sedentary households in the standard arm), mean small and large ruminants owned (more of both in the modified arm), and mean MUAC in children (lower in the standard arm).

### 3.4 Mediating factors for child undernutrition

#### 3.4.1 Beneficiaries

Table 3 presents the difference (modified minus standard arm) in the differences (endline minus baseline) for mediating factors for malnutrition among beneficiaries. There were no significant differences between arms at endline, with the exception of land access and ARI prevalence. There were, however, significant baseline to endline changes in several potentially important mediating factors. Household expenditure and food security improved for all beneficiaries; that is, falling household food insecurity access scores and coping strategies index scores, and rising diet diversity and food consumption scores. We also observed significant increases in land access and ruminant ownership. However, the prevalence of child sickness increased, because of a large increase in fever/malaria despite an increase in bed‐net use and a fall in ARI.

**Table 3:** Difference in differences for potential mediating factors of malnutrition among beneficiaries

	Endline minus baseline difference			Difference associated with the modified intervention^c^ (95% CI) *p* value
	Standard arm (June initiation)	Modified arm (April initiation)	Combined arms *p* value for endline minus baseline difference	
Clusters (*n*)^a^	10	10	20	
Households (*n*)^b^	1,030	885	1,915	
*Wealth*				
30‐day expenditure (GBP equivalent, mean ± *SD*)	5.97 ± 21.79	4.41 ± 21.22	5.25 ± 21.54 *p* < 0.001	−1.56 (−5.48, 2.35) .414
Access to land (% points, 95% CI)	1.7 (−0.5, 3.8)	6.6 (2.5, 10.7)	4.1 (1.6, 6.7) *p* < 0.001	5.0 (0.3, 9.6) .037
Large ruminants owned (mean ± *SD*)	0.1 ± 0.9	0.1 ± 1.1	0.1 ± 1.0 *p* < 0.001	−0.1 (−0.1, 0.0) .223
Small ruminants owned (mean ± *SD*)	0.1 ± 2.8	−0.1 ± 2.7	−0.0 ± 2.8 *p* < 0.001	−0.2 (−0.5, 0.1) .178
*Food security*				
Household food insecurity access score (mean ± *SD*) (0 best–27 worst)	−3.93 ± 6.51	−3.46 ± 6.84	−3.71 ± 6.67 *p* < 0.001	0.47 (−1.57, 2.50) .636
Coping strategies index score (mean ± *SD*) (0 best–56 worst)	−4.11 ± 10.83	−3.94 ± 11.04	−4.03 ± 10.93 *p* < 0.001	0.18 (−2.65, −3.00) .898
7‐day food consumption score (mean ± *SD*) (>35 “acceptable”)	5.68 ± 20.98	8.56 ± 19.80	7.01 ± 20.49 *p* < 0.001	2.88 (−1.45, 7.21) .181
24‐hr household dietary diversity score (mean ± *SD*) (min 0–max 12)	0.89 ± 2.61	1.18 ± 2.50	1.02 ± 2.56 *p* < 0.001	0.29 (−0.20, 0.78) .229
*Water and sanitation*				
Use of improved water source (% points, 95% CI)	3.5 (−11.2, 18.2)	−1.1 (−12.1, 9.9)	1.2 (−7.8, 10.2) *p* = 0.876	−4.6 (−23.0, 13.7) .603
Use of improved latrine (% points, 95% CI)	−1.0 (−3.2, 1.3)	−0.6 (−1.9, 0.7)	−0.8 (−2.0, 0.5) *p* = 0.455	0.4 (−2.2, 2.9) .777
*Children's infection and health behaviour*				
Sick in previous 4 weeks (% points, 95% CI)	12.7 (7.6, 17.8)	8.4 (−6.0, 22.8)	10.5 (3.0, 18.0) *p* = 0.014	−4.3 (−19.5, 11.0) .565
Sick with fever/malaria (men % points, 95% CI)	33.2 (23.7, 42.7)	36.0 (27.8, 44.1)	34.6 (28.4, 40.7) *p* < 0.001	2.7 (−9.8, 15.3) .652
Sick with diarrhoea (% points, 95% CI)	−4.3 (−11.0, 2.4)	−4.6 (−12.7, 3.5)	−4.5 (−9.6, 0.6) *p* = 0.096	−0.3 (−10.8, 10.2) .952
Sick with ARI (% points, 95% CI)	−8.5 (−15.6, 1.4)	−21.7 (−31.4, −12.1)	−15.1 (−21.8, −8.5) *p* < 0.001	−13.2 (−25.2, −1.2) .033
Slept under a mosquito net night before (% points, 95% CI)	79.3 (71.2, 87.5)	82.3 (76.9, 87.7)	80.8 (51.6, 94.3) *p* < 0.001	3.0 (−6.8, 12.8) .531
*Child diet diversity*				
Children 24–59 months old at baseline^d^ (*n*)	597	481	1,078	
Diet diversity (mean ± *SD*)	1.26 ± 1.73	1.23 ± 1.71	1.25 ± 1.72 *p* < 0.001	−0.03 (−0.59, 0.52) .904

*Note*. ARI = acute respiratory infection.

a Proportions analysed at cluster level.

b Means analysed at individual level.

c Difference in modified arm (initiated in April) minus standard arm (initiated in June).

d Children 30–67 months at endline.

We also performed cross‐sectional analyses to test for any difference in the prevalence of infant and young child feeding practices at endline; however, we found none, despite all indicators showing a trend towards improvement from baseline.

#### 3.4.2 Population sample

There were also no significant differences between the arms at endline among the population sample, with the exception of a significantly greater (though small) reduction in the size of small ruminant holdings in the modified arm (Table S3). Baseline to endline changes in the arms combined showed similar trends to those observed among beneficiaries.

**Figure 2:**
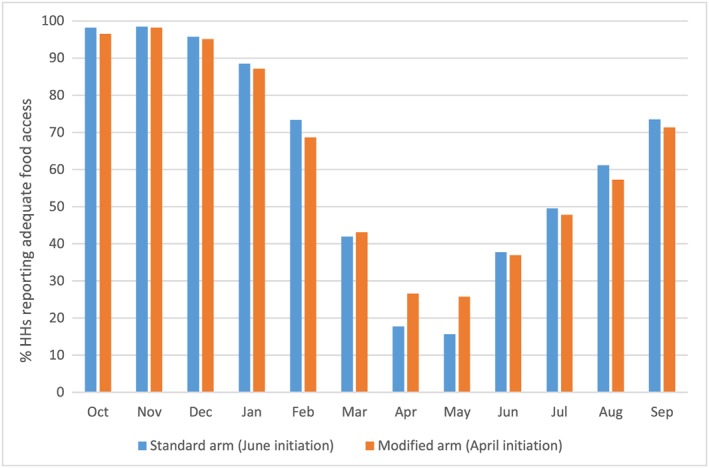
Months of adequate household food provisioning between October 2014 and September 2015 for cash beneficiaries, by trial arm, recalled from endline (October/November 2015)

To explore differences in food security over time, we examined recalled data on the months of adequate household food provisioning. A greater proportion of beneficiary households in the modified arm reported adequate food access in April and May (April 26.6% (95% CI [23.5, 29.8]), May 25.8% (95% CI [22.7, 29.0])) compared with beneficiaries in the standard arm who first received UCT in June (April 17.8% (95% CI [14.9, 21.0]), May 15.6% (95% CI [11.8, 20.5]); *p* < 0.001; Figure 2). However, there were no differences between the arms in any other months (*p* > 0.05).

### 3.5 Nutrition outcomes

We did not observe any difference in nutritional impact between the modified and standard interventions, either among beneficiary children (Table 4) or in the population (Table S4). The endline odds of a child having GAM were almost equal by arm, for both beneficiaries and the general population. The endline adjusted mean WHZ was also no different by arm in either sample. Stratifying by child age and household asset index did not reveal any differences. All other anthropometric outcomes were also no different by arm, both among beneficiaries and the population. We also observed no change in the prevalence of GAM in children 6–59 months old. Among beneficiaries aged 6–59 months, the GAM at baseline was 13.5% (95% CI [10.8, 16.8]) and at endline among beneficiary children of the same age it was 14.7% (95% CI [12.9, 16.9]), (*p* = 0.161). Within each arm, there were also no differences in GAM between baseline and endline (standard arm *p* = 0.426 and modified arm *p* = 0.231). Among children in the population sample, the baseline GAM was 13.2% (95% CI [10.3, 16.7]) and the endline GAM was 13.8% (95% CI [11.4, 16.6]; *p* = 0.590; standard arm *p* = 0.977 and modified arm *p* = 0.357).

**Table 4:** Odds ratios and coefficients for anthropometric outcomes among children in the modified arm (April initiation) compared with the standard arm (June initiation), among beneficiary households

Anthropometric outcome^a^	Model^b^^,^ c	Odds ratio (95% CI)	*P* value	Anthropometric outcome^a^	Model^b^^,^ c	Coefficient (95% CI)	*P* value
% global acute malnutrition	Crude endline global acute malnutrition (*n* = 1,690)	0.96 (0.67, 1.38)	0.826	Mean WHZ	Crude endline WHZ (*n* = 1,690)	0.04 (−0.10, 0.18)	0.573
	Adjusted for baseline WHZ, age, and sex (*n* = 1,671)	1.10 (0.77, 1.56)	0.601		Adjusted for baseline WHZ, age, and sex (*n* = 1,671)	−0.00 (−0.09 0.09)	0.984
	Adjusted for baseline WHZ, age, sex, lifestyle, land access, and baseline ARI (*n* = 1,671)	1.09 (0.77, 1.55)	0.630		Adjusted for baseline WHZ, age, sex, lifestyle, land access, and baseline ARI (*n* = 1,671)	0.00 (−0.09, 0.09)	0.93
% low MUAC (<125 mm)	Crude endline low MUAC (*n* = 1,749)	0.75 (0.42, 1.34)	0.338	Mean MUAC	Crude endline MUAC (*n* = 1,749)	0.23 (−1.99, 2.44)	0.842
	Adjusted for baseline MUAC, age, and sex (*n* = 1,732)	0.93 (0.42, 2.08)	0.862		Adjusted for baseline MUAC, age, and sex (*n* = 1,732)	−0.90 (−2.38, 0.58)	0.236
	Adjusted for baseline MUAC, age, sex, lifestyle, and baseline ARI (*n* = 1,732)	0.89 (0.39, 2.03)	0.773		Adjusted for baseline MUAC, age, sex, lifestyle, and baseline ARI (*n* = 1,732)	−0.85 (−2.24, 0.55)	0.236
% stunting (<−2 HAZ scores)	Crude endline stunting (*n* = 1,688)	0.98 (0.75, 1.30)	0.910	Mean HAZ	Crude endline HAZ (*n* = 1,688)	0.00 (−0.15, 0.15)	0.997
	Adjusted for baseline HAZ, age, and sex (*n* = 1,669)	1.33 (0.97, 1.84)	0.078		Adjusted for baseline HAZ, age, and sex (*n* = 1,669)	−0.04 (−0.09, 0.01)	0.153
	Adjusted for baseline HAZ, age, sex, lifestyle, land access, and baseline ARI (*n* = 1,669)	1.36 (0.98, 1.89)	0.066		Adjusted for baseline HAZ, age, sex, lifestyle, land access, and baseline ARI (*n* = 1,669)	−0.04 (−0.09, 0.02)	0.189

*Note*. WHZ = weight for height Z‐score; MUAC = mid‐upper arm circumference; HAZ = height for age Z‐score; ARI = acute respiratory infection.

a Among children who were 6–59 months old at baseline and 13–67 months old at endline.

b The model is a mixed effects multilevel logistic regression accounting for variation at the cluster and household level using random effects.

c The final models are only partially adjusted, as the two household level variables (household lifestyle and land access) had to be included at the individual level to allow the model to converge. The fully adjusted model did not converge.

Lastly, in order to investigate potential reasons for the lack of difference between the arms, as well as the failure of both interventions to reduce GAM, we analysed the association between recent sickness and the anthropometric status of beneficiary children aged 6–59 months. At both baseline and endline, children who had been sick within the last 30 days had a lower mean WHZ: −1.04 (95% CI [−1.21, −0.89]) compared with −0.88 (95% CI [−0.98, −0.78]; *p* = 0.011); and −1.11 (95% CI [−1.18, −1.04]) compared with −0.95 (95% CI [−1.08, −0.83]; *p* = 0.026), respectively. The prevalence of GAM among recently sick beneficiary children was also higher at baseline: sick: 17.9% (95% CI [13.6, 23.2]), compared with not sick: 11.7% (95% CI [5.2, 14.8]), *p* < 0.001; although not at endline, sick: 16.1% (13.1, 19.5), compared with not sick: 13.8% (95% CI [11.2, 17.0]), *p* = 0.322.

## 4 DISCUSSION

This is the first trial in a humanitarian context comparing two equal‐value seasonal cash interventions of different durations alongside supplementary feeding for PLW and children 6–<24 months in households receiving cash. It is also the first study to assess nutritional impact at population level, as well as among beneficiaries.

We found no differences in the endline prevalence of GAM, mean WHZ, or other anthropometric indicators, between children in households given cash from April compared with those given cash from June (among which, children 6–<24 months old also received supplementary food between June and September), and there was also no impact on the general population. The absence of a differential impact on endline anthropometry is consistent with the lack of differences in food security and health indicators between arms. It is interesting to note the temporary improvement in perceived food access among beneficiaries of the modified intervention in April and May compared with beneficiaries of the standard intervention, who during these 2 months received nothing. But evidently, this improvement in food security was insufficient to translate into a decreased risk of undernutrition by the end of the lean season. We did not test for differences in the prevalence of SAM as the study was not adequately powered to detect clinically important differences.

Unexpectedly, we found that the anthropometric status of children in households receiving either UCT or supplementary food, as well as other interventions and the harvest, was unchanged by endline and remained above the 10% emergency threshold (World Health Organization, 2000). This was surprising given prior studies in Niger that have documented postintervention/harvest improvements among beneficiary children (Aker & Nene, 2012; Bliss & Golden, 2013; Fenn et al., 2014).

### 4.1 Possible reasons for the lack of impact of the modified intervention

First, the smaller monthly transfers of the modified UCT may not have been sufficient; at 21,500FCFA/month (£24), they were a third less than the standard 32,500FCFA/month (£36) and were not accompanied by supplementary rations in April and May. Transfer size has been suggested as an important factor in determining the impact of cash on child outcomes (de Groot et al., 2015). However, analysis of expenditure data per capita indicated suggested that transfer size was not a limiting factor. Analysis of Concern's market monitoring data indicates that the UCT would have permitted purchase of food to meet between 88% (modified intervention) and 141% (standard intervention) of the average household's kilocalorie requirements, if solely spent on food and regardless of whether the household also received any supplementary food (Figures S1.3 and S1.4). A larger transfer may have increased spend on health‐seeking and thereby improved child nutritional status. This, however, seems unlikely given health service inaccessibility (Bailey & Hedlund, 2012; Blanford et al., 2012) and because barriers to uptake are rooted in chronic rather than transient poverty or seasonal food insecurity (Hampshire, Panter‐Brick, et al., 2009).

A second possible reason is that the UCT may not have been sufficiently early. Acute malnutrition admissions have been seen to rise from March onwards and we found more than half of households reporting food insecurity by the same month (Figure 2). Initiation of UCT prior to April has been suggested in Niger (Fenn et al., 2014) and some UCT interventions intended to prevent deterioration in food security and/or nutrition have even been implemented during/postharvest (Langendorf et al., 2014; Tumusiime, 2015).

### 4.2 Possible reasons neither intervention succeeded in reducing undernutrition

For both arms, there was a positive change over time in expenditure and food security. This was expected due to the harvest and is, plausibly, also attributable to the UCT and supplementary food as well as other, government‐led, interventions. These included distribution of 493 metric tonnes of grain in Affala and Takanamatt between April and September (with no discernible imbalance by arms, this was equivalent to the cereal needs of 22% of households in the studied villages for 6 months), and a social protection scheme providing 10,000FCFA/month (£11) to 840 households (also not discernibly different by arm, this was equivalent to 17% of households). As well as food availability, accessibility is also likely to have improved, particularly for beneficiary households for whom the transfers were theoretically enough to meet nearly all kilocalorie needs (Figure S1.4). Food utilisation also improved, as indicated by increased diet diversity and food consumption scores. As a backdrop, neither 2014 (CILSS et al., 2014) nor 2015 were crisis years for Tahoua department; the Integrated food security Phase Classification was Phase 2/stressed for 2015 (CILSS, 2015a, 2015b, 2015c).

However, although food security increased, we also saw a typical deterioration in health over the season. Among beneficiaries, child morbidity rose by 10% points by endline and of those who were sick, over 80% had fever/malaria, despite increased bed‐net use facilitated by seasonal distributions (insecticide‐treated bed‐net use at endline for children 6–59 months old was 97.4% (95% CI [96.0, 98.3])). A malaria peak is an annual norm, but the timing is perhaps later than typically characterised, with incidence remaining elevated several months after the rains, in early October (Fenn et al., 2014; Vaitla, Devereux, & Swan, 2009; Figure S1.5). It is also worth noting that improved latrines were accessible to only 2% of households, 35% did not use improved water sources and these indicators remained unchanged following receipt of either UCT. As is typical among children from UCT beneficiary households in Niger (Bliss et al., 2016; Fenn et al., 2014), we found a significant association between recent sickness and lower mean WHZ and higher GAM among beneficiary children. Infections, including malaria, are important determinants of malnutrition (Black et al., 2008; Bliss et al., 2016) and may have limited the effectiveness of *both* UCTs and the 4 months of supplementary feeding to prevent acute malnutrition in 2015 (Bliss et al., 2016; Fenn et al., 2014; Save the Children, 2009).

### 4.3 Strengths and limitations

Strengths of our study include the cluster‐randomised controlled trial design and high response and follow‐up rates of our cohort. We aimed for a sample size of 3,520 beneficiary children but found only 1,831 (52.0%), due to an overestimation of average household size and number of children per household. However, we also found a much lower prevalence of baseline GAM than we had anticipated (14% vs. 21%). A post hoc calculation using the clustersampsi command in Stata 14 indicates that, with 80% power and an alpha risk of 5%, we would have been able to detect a difference in endline GAM prevalence of 6% points with both our achieved samples of 1,831 beneficiary children and the combined sample of 2,292 beneficiary and nonbeneficiary children. Although the achieved sample size was less than planned, our results indicate that the modified intervention did not result in any major differences in the prevalence of GAM. Limitations are that we did not measure the incidence of outcomes during the intervention to understand any transitory impacts, particularly in the first 2 months of the modified UCT, and the limited generalisability of findings to periods of acute food insecurity, given the absence of crisis indicators during 2015.

To conclude, although we observed a temporary increase in food security for beneficiaries in the pre‐lean season, there was no evidence that starting the UCT 2 months earlier and providing the same amount of cash over a longer period, together with 4 months of supplementary feeding, would be beneficial to children's nutritional status. There is already consensus that cash usually needs to be combined with complementary interventions to impact on nutrition (Bailey & Hedlund, 2012; de Groot et al., 2015). Probably because past recommendations have focused heavily on the food security‐related drivers of malnutrition (Vaitla et al., 2009), the UCT combined with supplementary food has become the standard lean season intervention in Niger (Langendorf et al., 2014). However, the potential positive effects of UCT on undernutrition can be limited by prevailing poor health (Bliss et al., 2016; Fenn et al., 2014; Save the Children, 2009) and there is a pre‐existing recommendation that the UCT/supplementary food package is implemented where health needs are met (Langendorf et al., 2014). We observed an elevated prevalence of GAM in both arms at baseline and endline, despite improved food security, and there was an association between nutritional status and infection. Therefore, in the future, it would be pertinent to assess any supply‐side shortcomings in the health system and barriers to health‐seeking, as well as availability and barriers to use of safe water and latrines, in addition to ensuring household food security. More specifically, we suggest that seasonal interventions to treat and prevent malaria, a major cause of death (Langendorf et al., 2014) and an important driver of acute malnutrition in this population (Bliss et al., 2016), in addition to the standard UCT/nutrition supplement intervention, may help protect children from acute malnutrition. Lastly, it is also recommended that future studies test the assumption that the targeting of low income households with UCTs leads to population level impact (de Groot et al., 2015).

## CONFLICTS OF INTERESTS

The authors declare that they have no conflicts of interest.

## CONTRIBUTIONS

VS, CG‐E, AS, JM, and JL conceived and designed the study. VS wrote the first draft of the manuscript. VS, CG‐E, KK, JL, HH‐B, JS‐W, TC, JM, and AS contributed edits to the manuscript. VS, CG‐E, GN, KK, and AS supervised the trial. CG‐E managed the data. VS, CG‐E, HH‐B, and TC analysed the data.

## Supporting information

Fig. S1.1 Admissions of children (6–59 months old) with uncomplicated severe acute malnutrition to outpatient treatment programmes in Tahoua department in 2013
Figure S1.2 Seasonal calendar for Tahoua, Niger
Figure S1.3 Average selling prices (/FCFA) of key commodities between April and November 2015, in five markets in Affala and Takanamatt, Tahoua, Niger (*n* = number of market visits at which data were collected; 1 tia millet/sorghum = 2.5 kg and 1 tia cowpea = 2.8 kg, 1 tia millet/sorghum = 1 days consumption for household of 5 and
Figure S1.4 Percentage of the standard food basket of rations for a household of 5 people covered by the monthly cash transfer in 2015, by trial arm
Figure S1.5 Cases of malaria reported in children by epidemiological week between March and December 2015 in health centres in Affala and Takanamatt, Tahoua, Niger (diagnosed with rapid diagnostic tests or by symptoms when test kits were not available)

Fig. S2.1 Flow diagram for participating households

**Table S1.** Baseline characteristics of population sample households (beneficiaries and non‐beneficiaries in targeted villages)

**Table S2.** Baseline characteristics of population sample children (beneficiaries and non‐beneficiaries in targeted villages)

**Table S3.** Difference in differences for potential mediating factors of malnutrition among the population sample (beneficiaries and non‐beneficiaries in targeted villages)

**Table S4.** Odds ratios and regression coefficients for nutrition outcomes among children in the modified arm (April initiation) compared to the standard arm (June initiation), among the population sample (beneficiaries and non‐beneficiaries in targeted villages)
