# Supplementary material for: Findings from a cluster randomised trial of unconditional cash transfers in Niger
Source: Matern Child Nutr. 2018 May 8;14(4):e12615. doi: 10.1111/mcn.12615 (PMC6175357; doi:10.1111/mcn.12615)
Supplement: Supplementary file 1 — Fig. S1.1 Admissions of children (6–59 months old) with uncomplicated severe acute malnutrition to outpatient treatment programmes in Tahoua department in 2013 Figure S1.2 Seasonal calendar for Tahoua, Niger Figure S1.3 Average selling prices (/FCFA) of key commodities between April and November 2015, in five markets in Affala and Takanamatt, Tahoua, Niger (n = number of market visits at which data were collected; 1 tia millet/sorghum = 2.5 kg and 1 tia cowpea = 2.8 kg, 1 tia millet/sorghum = 1 days consumption for household of 5 and Figure S1.4 Percentage of the standard food basket of rations for a household of 5 people covered by the monthly cash transfer in 2015, by trial arm Figure S1.5 Cases of malaria reported in children by epidemiological week between March and December 2015 in health centres in Affala and Takanamatt, Tahoua, Niger (diagnosed with rapid diagnostic tests or by symptoms when test kits were not available) [file MCN-14-e12615-s001.pdf]

Appendix 1

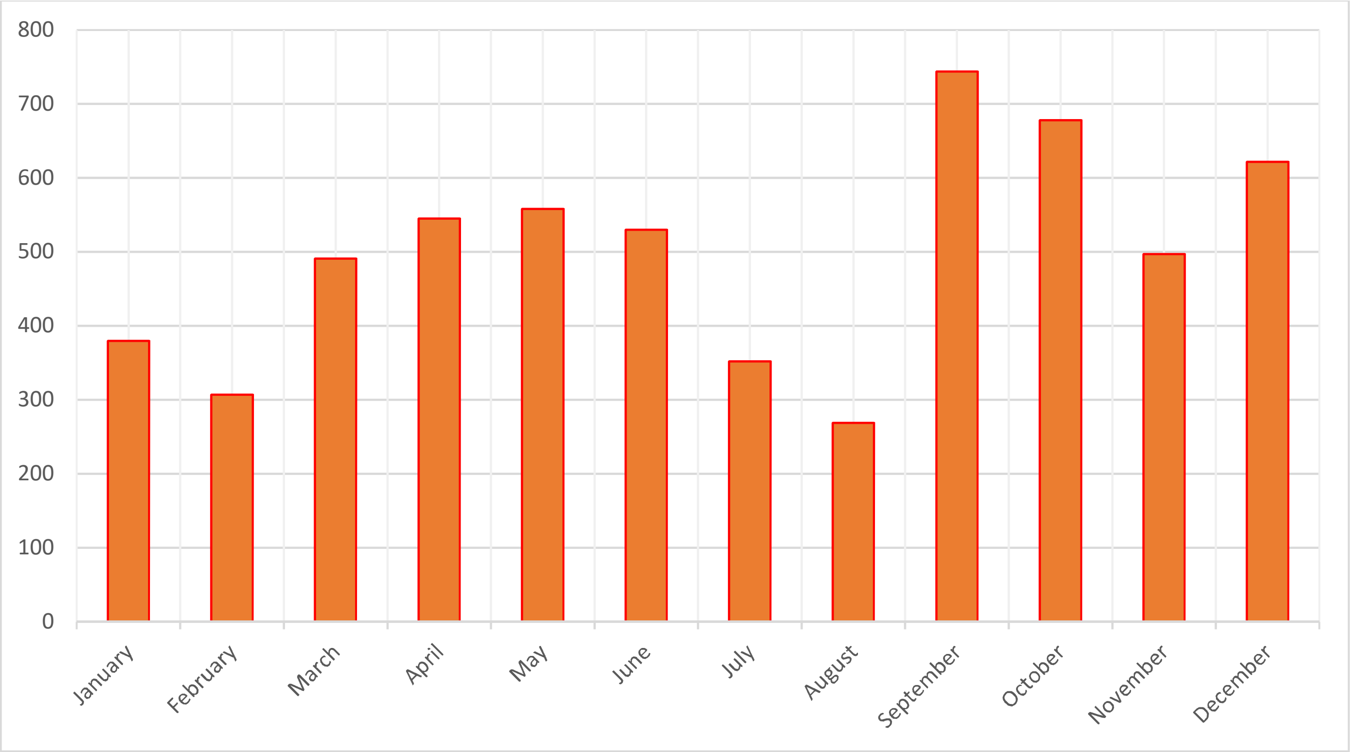

Fig. 1.1 Admissions of children (6-59 months old) with uncomplicated severe acute malnutrition to outpatient treatment programmes in Tahoua department in 2013

Month	Jan	Feb	Mar	Apr	May	Jun	Jul	Aug	Sep	Oct	Nov	Dec
Rains						X	X	X	X			
**Agricultural activities**												
Millet			Land preparation			Plant			Eat green	Harvest		Sell
Sorghum	Sell		Land preparation			Plant					Harvest	
Cow pea	Sell		Land preparation				Plant		Harvest			Sell
Market gardening	Cultiv-ate	Sell			No gardening tasks					Grow seed-lings	Garden preparation	
**Livestock**												
Animal migration	At home			Leave	Away				Return	At home		
Animal sales	Buy & sell	Sell								Buy		
Labour migration	Away		Return						Leave			
**Disease**												
Malaria						X	X	X	X	X	X	

Fig. 1.2 Seasonal calendar for Tahoua, Niger

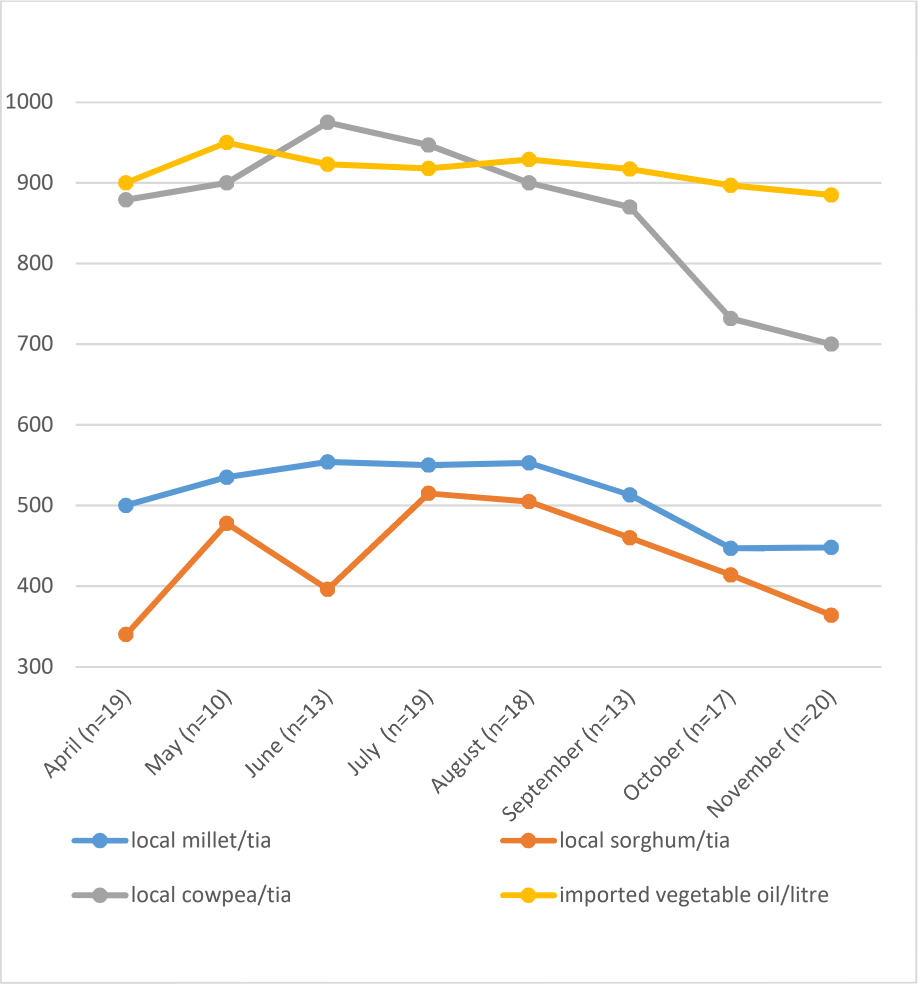

Fig. 1.3 Average selling prices (/FCFA) of key commodities between April and November 2015, in five markets in Affala and Takanamatt, Tahoua, Niger
(n = number of market visits at which data were collected;
1 tia millet/sorghum = 2.5kg and 1 tia cowpea = 2.8kg,
1 tia millet/sorghum=1 days consumption for household of 5 and

1 tia cowpea = 5.5 days for a household of 5, according to the WFP's standard ration of 500g of cereal and 100g of pulses per person per day)

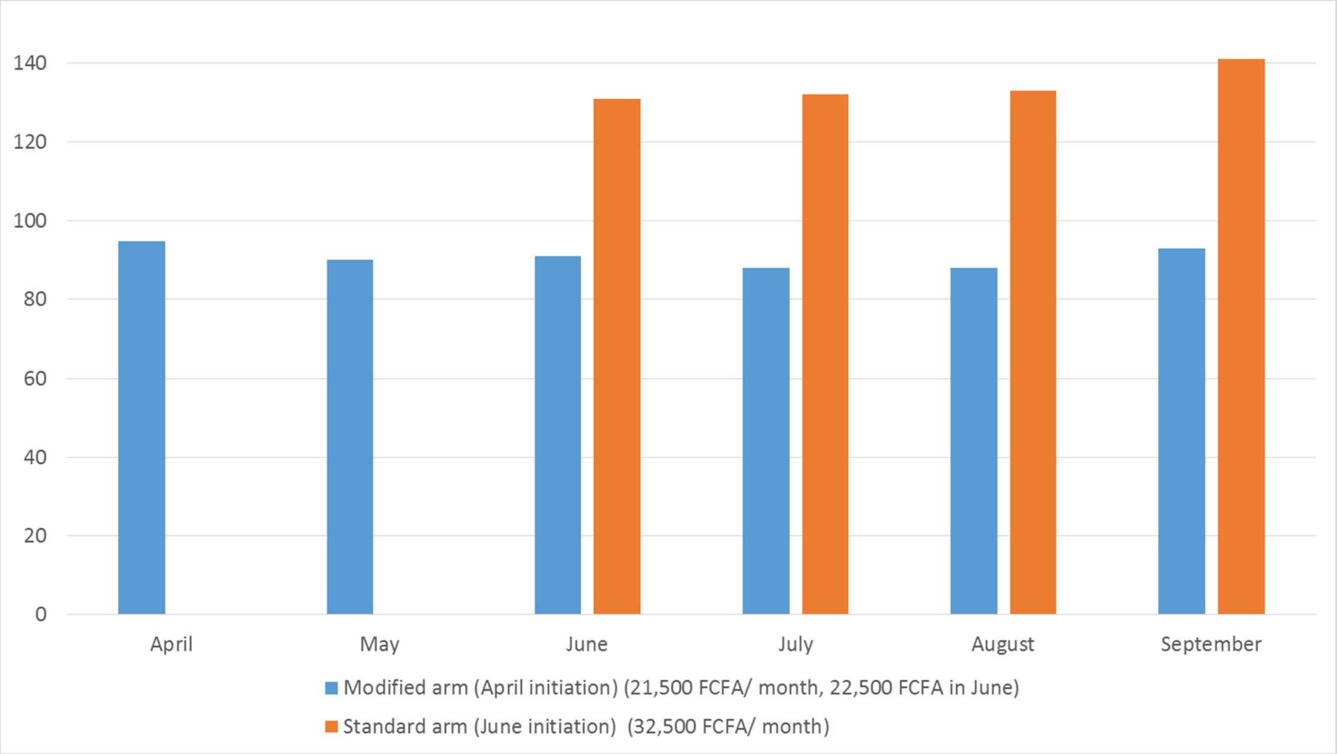

Fig. 1.4 Percentage of the standard food basket of rations for a household of 5 people covered by the monthly cash transfer in 2015, by trial arm

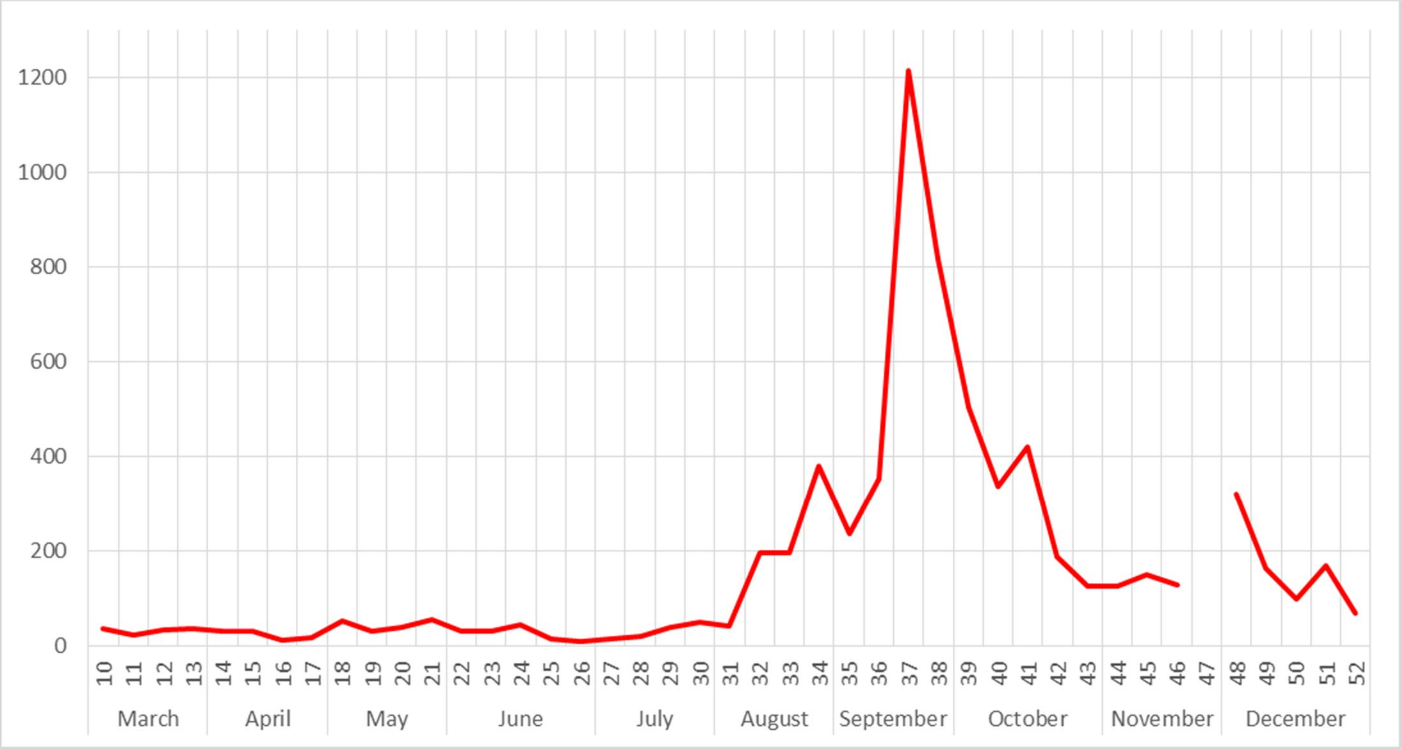

Fig. 1.5 Cases of malaria reported in children by epidemiological week between March and December 2015 in health centres in Affala and Takanamatt, Tahoua, Niger (diagnosed with rapid diagnostic tests or by symptoms when test kits were not available)
