# Supplementary figures and images for: Findings from a cluster randomised trial of unconditional cash transfers in Niger
Source: Matern Child Nutr. 2018 May 8;14(4):e12615. doi: 10.1111/mcn.12615 (PMC6175357; doi:10.1111/mcn.12615)

**Appendix 2**


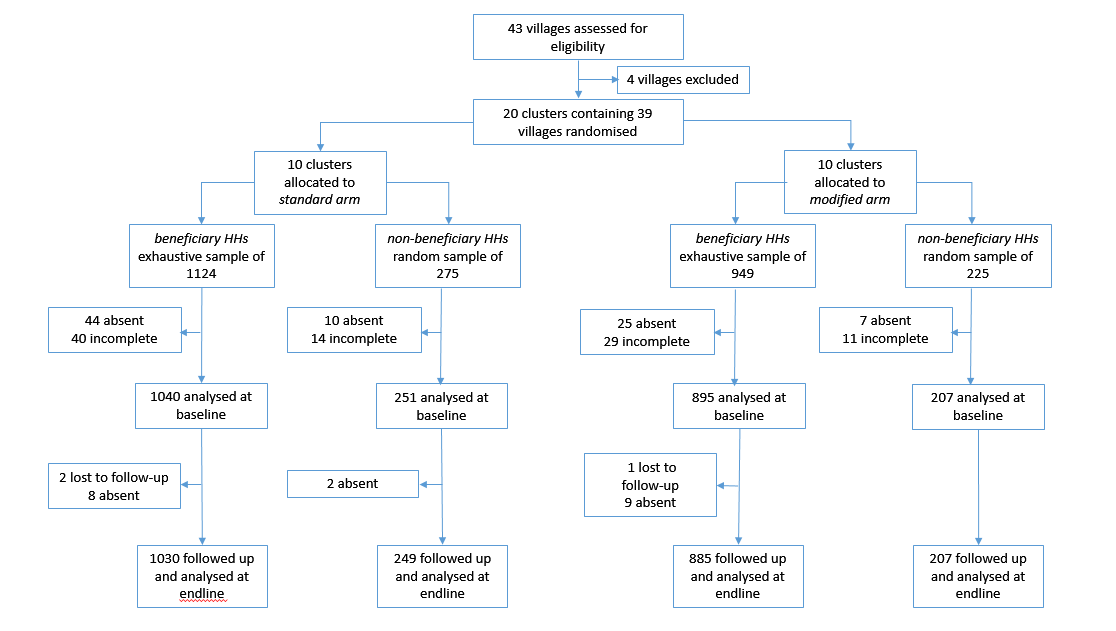


Fig. 2.1 Flow diagram for participating households

Supplement: Supplementary file 2 — Fig. S2.1 Flow diagram for participating households [file MCN-14-e12615-s002.docx]
