# Supplementary material for: Findings from a cluster randomised trial of unconditional cash transfers in Niger
Source: Matern Child Nutr. 2018 May 8;14(4):e12615. doi: 10.1111/mcn.12615 (PMC6175357; doi:10.1111/mcn.12615)
Supplement: Supplementary file 3 — Table S1. Baseline characteristics of population sample households (beneficiaries and non‐beneficiaries in targeted villages) [file MCN-14-e12615-s003.docx]

**Appendix 3:**

**Table 1.** Baseline characteristics of population sample households (beneficiaries and non-beneficiaries in targeted villages)

| Characteristic |  | Standard arm (June initiation) | Modified arm (April initiation) | Combined arms | *P* value |
| --- | --- | --- | --- | --- | --- |
| **Households (n)** | | 1291 | 1102 | 2393 |  |
| *Sociodemographic characteristics* | | | | |  |
| Number in HH (mean ± SD) | | 6.4 ± 3.3 | 6.1 ± 3.0 | 6.3 ± 3.2 | *P*=0.389 |
| Ethnicity of HH head (n [%] 95% CI) | Hausa | 966 (76.0%)  (58.4, 87.7) | 609 (60.8%)  (45.4, 74.4) | 1575 (69.1%)  (56.7, 79.2) | *P*=0.156 |
|  | Tuareg | 303 (22.5%)  (11.0, 40.6%) | 455 (35.8%)  (23.0, 50.9) | 758 (28.6%)  (18.9, 40.8) |  |
|  | Fulani/Peulh | 18 (1.1%)  (0.5, 2.4) | 37 (3.4%)  (1.6, 7.1) | 55 (2.1%)  (1.1, 4.1) |  |
|  | Other | 4 (0.4%)  (0.1, 1.2) | 1 (0.1%)  (0.0, 0.3) | 5 (0.2%)  (0.1, 0.7) |  |
| Sex of HH head (n [%] 95% CI) | Male | 1018 (82.5%)  (77.8, 86.3) | 820 (77.3%)  (71.6, 82.2) | 1838 (80.1%)  (76.3, 83.5) | *P*=0.127 |
| Lifestyle of HH (n [%] 95% CI) | Sedentary | 1140 (87.6%)  (76.2, 94.0) | 1066 (96.5%)  (94.4, 97.8) | 2206 (91.6%) (84.8, 95.6) | *P*=0.005 |
|  | Nomad | 81 (7.8%)  (3.1, 18.3) | 30 (2.8%)  (1.8, 4.3) | 111 (5.5%) (2.6, 11.3) |  |
|  | Transhumant/  Other | 70 (4.6%)  (2.2, 9.3) | 6 (0.7%)  (0.2, 2.5) | 76 (2.8%)  (1.4, 5.7) |  |
| *Wealth* | | | | | |
| 30 day expenditure (GBP equivalent, mean ± SD, range) | | 33.78 ± 23.98  (0 – 178.97) | 34.88 ± 23.09  (0.55 –131.87) | 34.29 ± 23.59  (0 – 178.97) | *P*=0.615 |
| Access to land (n [%] 95% CI) | | 1240 (96.0) (93.5, 97.6) | 1034 (95.4) (92.7, 97.1) | 2274 (95.7) (94.0, 97.0) | *P*=0.636 |
| Large ruminants owned (mean ± SD, range) | | 0.7 ± 1.2  (0 - 13) | 1.1 ± 3.0  (0 – 52) | 0.9 ± 2.2  (0 – 52) | *P*=0.033^a^ |
| Small ruminants owned (mean ± SD, range) | | 2.6 ± 3.5  (0 - 36) | 3.4 ± 3.7  (0 - 20) | 3.0 ± 3.6  (0 - 36) | *P*=0.005^a^ |
| *Food security* | | | | | |
| Household Food Insecurity Access score (mean ± SD, range) (0 best – 27 worst) | | 6.2 ± 5.1 | 6.2 ± 5.2 | 6.2 ± 5.2 | *P*=0.965 |
| Coping Strategies Index score (mean ± SD, range) (0 best – 56 worst) | | 7.1 ± 8.9 | 6.8 ± 8.6 | 6.9 ± 8.7 | *P*=0.588 |
| 7 day Food Consumption Score (mean ± SD, range) (>35 ‘acceptable’) | | 41.4 ± 17.3  (3 – 104.0) | 45.7 ± 16.1  (6.5 – 101.0) | 43.4 ± 16.9  (3 – 104.0) | *P*=0.094 |
| 24 hour Household Dietary Diversity Score^b^ (mean ± SD) (min 0 – max 12) | | 4.0 ± 2.0 | 4.4 ± 2.0 | 4.2 ± 2.0 | *P*=0.214 |
| *Sanitation and hygiene* | | | | | |
| Use of improved water source (n [%] 95% CI) | | 935 (70.2%) (54.8, 82.1) | 635 (56.9%) (45.4, 67.7) | 1570 (64.1%)  (53.8, 73.3) | *P*=0.148 |
| Use of improved latrine (n [%] 95% CI) | | 23 (1.9%)  (0.7, 5.2) | 27 (2.0%)  (0.6, 6.8) | 50 (1.9%)  (0.9, 4.3) | *P*=0.948 |

^a^ result of test on square root transformed data

^b^ denominator for modified arm: 1101
