# Supplementary material for: Findings from a cluster randomised trial of unconditional cash transfers in Niger
Source: Matern Child Nutr. 2018 May 8;14(4):e12615. doi: 10.1111/mcn.12615 (PMC6175357; doi:10.1111/mcn.12615)
Supplement: Supplementary file 4 — Table S2. Baseline characteristics of population sample children (beneficiaries and non‐beneficiaries in targeted villages) [file MCN-14-e12615-s004.docx]

**Appendix 3:**

**Table 2.** Baseline characteristics of population sample children (beneficiaries and non-beneficiaries in targeted villages)

| Characteristic | Standard arm (June initiation) | Modified arm (April initiation) | Combined arms | *P* value |
| --- | --- | --- | --- | --- |
| **Children (n)** | 1268 | 1024 | 2292 |  |
| Male sex (n [%] 95% CI) | 612 (45.4%)  (40.7, 50.2) | 512 (49.5%)  (44.7, 54.4) | 1124 (47.2%)  (43.8, 50.7) | *P*=0.218 |
| Age (months, mean ± SD) | 30.2 ± 13.5 | 31.2 ± 14.3 | 30.6 ± 13.9 | *P*=0.299 |
| *Nutrition status* | | | | |
| Weight-for-height (Z-score, mean ± SD)^a^ | -0.94 ± 1.01 | -0.85 ± 0.97 | -0.9 ± 0.99 | *P*=0.329 |
| Wasted (<-2 WHZ, n [%] 95% CI)^a^ | 169 (14.1%)  (9.6, 20.3) | 120 (11.5%)  (9.3, 14.0) | 289 (12.9%)  (10.0, 16.5) | *P*=0.325 |
| Global Acute Malnutrition (<-2 WHZ and/or oedema, n [%] 95% CI)^b^ | 173 (14.4%)  (10.0, 20.4) | 122 (11.6%)  (9.4, 14.1) | 295 (13.2%)  (10.3, 16.7) | *P*=0.278 |
| MUAC (mm, mean ± SD)^c^ | 143 ± 12 | 145 ± 12 | 144 ± 12 | *P*=0.042 |
| Low MUAC (<125mm, n [%] 95% CI)^c^ | 88 (6.5%)  (4.7, 9.0) | 40 (4.2%)  (2.7, 6.4) | 128 (5.5%)  (4.1, 7.2) | *P*=0.099 |
| Height-for-age (Z-score, mean ± SD)^d^ | -1.38 ± 1.37 | -1.43 ± 1.27 | -1.40 ± 1.33 | *P*=0.665 |
| Stunted (<-2 HAZ, n [%] 95% CI)^d^ | 431 (33.5%)  (28.9, 38.3) | 329 (31.6%)  (26.9, 36.7) | 760 (32.6%)  (29.2, 36.2) | *P*=0.567 |
| *Infection and health behaviour ^e^* | | | | |
| Sick in previous 4 weeks (n [%] 95% CI) | 378 (34.2%)  (24.5, 45.4) | 330 (33.8%)  (26.9, 41.5) | 708 (34.0%)  (27.7, 41.0) | *P*=0.951 |
| Sick with fever/malaria (n [%] 95% CI) | 219 (58.3%)  (50.0, 66.2) | 178 (55.1%)  (47.5, 62.4) | 397 (56.9%)  (51.1, 62.5) | *P*=0.550 |
| Sick with diarrhoea (n [%] 95% CI) | 72 (19.7%)  (15.8, 24.3) | 64 (20.0%)  (15.6, 25.2) | 136 (19.8%)  (16.8, 23.2) | *P*=0.924 |
| Sick with ARI (n [%] 95% CI) | 91 (26.4%)  (16.6, 39.2) | 112 (32.0%)  (23.4, 42.0) | 203 (28.9%)  (21.8, 37.2) | *P*=0.442 |
| Slept under a mosquito net night before (n [%] 95% CI) | 118 (8.4%)  (6.1, 11.4) | 87 (7.9%)  (3.8, 15.7) | 205 (8.2%)  (5.7,11.6) | *P*=0.872 |
| *Care/nutrient intake* | | | | |
| **Children 6-<24 months** | 472 | 379 | 851 |  |
| Ever breastfed (n [%] 95% CI) | 413 (89.1%) (85.2, 92.1) | 319 (84.7%) (77.1, 90.1) | 732 (87.1%  (82.9, 90.4) | *P*=0.192 |
| Continued breastfeeding at 1 year (n [%] 95% CI) ^f^ | 88 (85.7%)  (69.1, 94.2) | 72 (83.0%)  (70.8, 90.8) | 160 (84.6%)  (74.7, 91.0) | *P*=0.726 |
| Minimum dietary diversity (n [%] 95% CI) | 75 (15.6%) (10.4, 22.8) | 80 (20.2%) (14.5, 27.4) | 155 (17.7%)  (13.4, 22.9) | *P*=0.294 |
| Minimum meal frequency (n [%] 95% CI) | 111 (29.9%) (22.0, 39.2) | 89 (25.5%) (17.1, 36.2) | 200 (27.9%)  (21.9, 34.8) | *P*=0.493 |
| Minimum adequate diet (n [%] 95% CI) | 19 (5.6%)  (2.7, 11.3) | 22 (5.8%)  (2.4, 13.6) | 41 (5.7%)  (3.2, 9.9) | *P*=0.937 |
| **Children 24-<59 months** | 796 | 645 | 1441 |  |
| 7 food group diet diversity score (mean ± SD) | 2.4 ± 1.2 | 2.5 ± 1.4 | 2.4 ± 1.3 | *P*=0.593 |

^a^ denominator for standard arm: 1216, and for modified arm: 978

^b^ denominator for standard arm: 1220, and for modified arm: 980; there were 6 oedema cases in total, 4 in the standard arm and 2 in the modified arm

^c^ denominator for standard arm: 1255, and for modified arm: 1014

^d^ denominator for standard arm: 1214, and for modified arm: 976

^e^ denominator for modified arm: 1023

^f^ estimated for children 12-15 months only; denominator for standard arm: 105, and for modified arm: 86
