# Supplementary material for: Findings from a cluster randomised trial of unconditional cash transfers in Niger
Source: Matern Child Nutr. 2018 May 8;14(4):e12615. doi: 10.1111/mcn.12615 (PMC6175357; doi:10.1111/mcn.12615)
Supplement: Supplementary file 5 — Table S3. Difference in differences for potential mediating factors of malnutrition among the population sample (beneficiaries and non‐beneficiaries in targeted villages) [file MCN-14-e12615-s005.docx]

**Appendix 3**

**Table 3.** Difference in differences for potential mediating factors of malnutrition among the population sample (beneficiaries and non-beneficiaries in targeted villages)

|  | Endline minus baseline difference | | |  |
| --- | --- | --- | --- | --- |
|  | Standard arm (June initiation) | Modified arm (April initiation) | Combined arms  *P* value for endline minus baseline difference | Difference associated with the modified intervention^c^  (95% CI)  *P* value |
| Clusters (n)^a^ | 10 | 10 | 20 |  |
| Households (n)^b^ | 1279 | 1092 | 2371 |  |
| *Wealth* | | | | |
| 30 day expenditure (GBP equivalent, mean ± SD) | 3.75 ± 26.65 | 3.11 ± 26.24 | 3.45 ± 26.47  *P*<0.001 | -0.64  (-4.16, 2.87)  *P*=0.706 |
| Access to land (mean % points ± SD) | 2.25 ± 3.10 | 3.56 ± 5.35 | 2.91 ± 4.30  *P*=0.005 | 1.31  (-2.78, 5.39)  *P*=0.511 |
| Large ruminants owned (mean ± SD) | 0.1 ± 1.1 | -0.1 ± 2.9 | 0.0 ± 2.1  *P*=0.805 | -0.2  (-0.5, 0.1)  *P*=0.222 |
| Small ruminants owned (mean ± SD) | -0.1 ± 3.5 | -0.6 ± 3.9 | -0.3 ± 3.7  *P*=0.021 | -0.5  (-0.9, -0.1)  *P*=0.017 |
| *Food security* | | | | |
| Household Food Insecurity Access score (mean ± SD) (0 best – 27 worst) | -2.84 ± 6.43 | -2.29 ± 6.48 | -2.59 ± 6.46  *P*<0.001 | 0.55  (-1.61, 2.71)  *P*=0.600 |
| Coping Strategies Index score (mean ± SD) (0 best – 56 worst) | -3.0 ± 10.4 | -1.7 ± 10.5 | -2.4 ± 10.5  *P*<0.001 | 1.33  (-1.1, 3.8)  *P*=0.266 |
| 7 day Food Consumption Score (mean ± SD) (>35 ‘acceptable’) | 6.7 ± 20.7 | 8.9 ± 20.1 | 7.7 ± 20.5  *P*<0.001 | 2.2  (-2.8, 7.2)  *P*=0.361 |
| 24 hour Household Dietary Diversity Score (mean ± SD) (min 0 – max 12) | 0.95 ± 2.54 | 1.11 ± 2.49 | 1.02 ± 2.52  *P*<0.001 | 0.16  (-0.47 – 0.79)  *P*=0.609 |
| *Water and sanitation* | | | | |
| Use of improved water source (mean % points ± SD) | -0.29 ± 20.88 | 5.09 ± 15.88 | 2.40 ± 18.27  *P*=0.746 | -5.38  (-11.99, 22.75)  *P*=0.524 |
| Use of improved latrine (mean % points ± SD) | -0.68 ± 2.17 | -1.17 ± 1.98 | -0.92 ± 2.03  *P*=0.276 | -0.49  (-2.43, 1.46)  *P*=0.606 |
| *Children’s infection and health behaviour* | | | | |
| Sick in previous 4 weeks (mean % points ± SD) | 7.96 ± 9.83 | 9.46 ± 19.36 | 8.71 ± 14.96  *P*=0.048 | 1.50  (-12.87, 15.87)  *P*=0.830 |
| Sick with fever/malaria (mean % points ± SD) | 27.85 ± 19.65 | 30.48 ± 15.85 | 29.17 ± 17.43  *P*<0.001 | 2.63  (-14.07, 19.34)  *P*=0.745 |
| Sick with diarrhoea (mean % points ± SD) | -4.18 ± 13.01 | -8.85 ± 12.43 | -6.52 ± 12.61  *P*<0.016 | -4.67  (-16.58, 7.34)  *P*=0.422 |
| Sick with ARI (mean % points ± SD) | -7.73 ± 20.87 | -14.51 ± 19.74 | -11.12 ± 20.08  *P*<0.012 | -6.78  (-25.80, 12.23)  *P*=0.464 |
| Slept under a mosquito net night before (mean % points ± SD) | 80.46 ± 11.00 | 81.99 ± 10.40 | 81.22 ± 10.45  *P*<0.001 | 1.53  (-8.49, 11.55)  *P*=0.753 |
| *Child diet diversity* | | | | |
| Children 24-59 months old at baseline^d^ (n) | 765 | 615 | 1380 |  |
| Diet diversity (mean ± SD) | 1.22 ± 1.80 | 1.22 ± 1.77 | 1.22 ± 1.78  *P*<0.001 | -0.00  (-0.55, 0.55)  *P*=0.997 |

^a^ proportions analysed at cluster level

^b^ means analysed at individual level

^c^ difference in modified arm (initiated in April) minus standard arm (initiated in June)

^d^ children 30-67 months at endline
