# Supplementary material for: Findings from a cluster randomised trial of unconditional cash transfers in Niger
Source: Matern Child Nutr. 2018 May 8;14(4):e12615. doi: 10.1111/mcn.12615 (PMC6175357; doi:10.1111/mcn.12615)
Supplement: Supplementary file 6 — Table S4. Odds ratios and regression coefficients for nutrition outcomes among children in the modified arm (April initiation) compared to the standard arm (June initiation), among the population sample (beneficiaries and non‐beneficiaries in targeted villages) [file MCN-14-e12615-s006.docx]

**Appendix 3:**

**Table 4.** Odds ratios and regression coefficients for nutrition outcomes among children in the modified arm (April initiation) compared to the standard arm (June initiation), among the population sample (beneficiaries and non-beneficiaries in targeted villages)

| Nutrition Outcome^a^ | Model^b^ | Odds Ratio | *P* value | Nutrition Outcome^a^ | Model^b^ | Coefficient | *P* value |
| --- | --- | --- | --- | --- | --- | --- | --- |
| % Global Acute Malnu-trition | Crude endline Global Acute Malnutrition  (n=2112)^c^ | 0.70  (0.48, 1.02) | *P*=0.061 | Mean Weight for Height Z-Score | Crude endline weight for height Z-score  (n=2112) | 0.04  (-0.12, 0.19) | *P*=0.638 |
|  | Adjusted for baseline WHZ, age and sex  (n=2091)^d^ | 0.93  (0.62, 1.40) | *P*=0.728 |  | Adjusted for baseline WHZ, age and sex (n=2091) | 0.01  (-0.08, 0.10) | *P*=0.855 |
|  | Adjusted for baseline WHZ, age, sex, lifestyle and small ruminant holdings  (n=2091)^e^ | 0.93  (0.58, 1.49) | *P*=0.759 |  | Adjusted for baseline WHZ, age, sex, lifestyle and small ruminant holdings (n=2091) | 0.01  (-0.08, 0.10) | *P*=0.849 |
| % low MUAC (<125  mm) | Crude endline low MUAC  (n=2187) | 0.49  (0.10, 2.28) | *P*=0.360 | Mean MUAC | Crude endline MUAC  (n=2187) | 0.36  (-1.82, 2.54) | *P*=0.748 |
|  | Adjusted for baseline MUAC, age and sex  (n=2167) | 0.64  (0.25, 1.63) | *P*=0.344 |  | Adjusted for baseline MUAC, age and sex (n=2167) | -0.69  (-2.13, 0.74) | *P*=0.345 |
|  | Adjusted for baseline MUAC, age, sex, lifestyle and small ruminant holdings  (n=2167) | 0.71  (0.27, 0.18) | *P*=0.477 |  | Adjusted for baseline MUAC, age, sex, lifestyle and small ruminant holdings (n=2167) | -0.66  (-2.12, 0.80) | *P*=0.375 |
| % stunting (<-2 HAZ scores) | Crude endline stunting  (n=2109) | 1.11  (0.66, 1.87) | *P*=0.696 | Mean Height for Age Z-Score | Crude endline height for age Z-score  (n=2109) | -0.66  (-0.23, 0.11) | *P*=0.496 |
|  | Adjusted for baseline HAZ, age and sex  (n=2088) | 1.51  (0.96, 2.38) | *P*=0.072 |  | Adjusted for baseline HAZ, age and sex  (n=2088) | -0.04  (-0.09, 0.01) | *P*=0.086 |
|  | Adjusted for baseline HAZ, age, sex, lifestyle and small ruminant holdings  (n=2088) | 1.53  (0.98, 2.37) | *P*=0.059 |  | Adjusted for baseline HAZ, age, sex, lifestyle and small ruminant holdings (n=2088) | -0.04  (-0.09, 0.01) | *P*=0.111 |

^a^ Among children who were 6-59 months old at baseline and 13-67 months old at endline

^b^ All analyses are adjusted for clustering at household and cluster level unless otherwise specified

^c^ This model is adjusted for clustering at cluster level only because the model does not converge when household level is included.

^d^ For comparison with the prior model, the odds ratio for this model adjusted for clustering at cluster level only is 0.71 (95% CI 0.46, 1.10), *P*=0.129

^e^ For comparison with the prior model, the odds ratio for this model adjusted for clustering at cluster level only is 0.76 (95% CI 0.47, 1.20), *P*=0.238
